# P-524. Comparative Costs of Implementing an Evidence-Based Online HIV Prevention Program: Direct-to-Consumer Marketing vs. Community-Based Organization Recruitment

**DOI:** 10.1093/ofid/ofae631.723

**Published:** 2025-01-29

**Authors:** Sarah J Munroe, Bruce R Schackman, Krystal Madkins, Rana Saber, Kathryn Macapagal, Jocelyn Vititow, Nicholas Sweeney, Noah Feder, Nanette Benbow, Brian Mustanski, Benjamin P Linas

**Affiliations:** Boston Medical Center, Gloucester, Massachusetts; Weill Cornell Medicine, New York, New York; Northwestern University, Chicago, Illinois; Northwestern University, Chicago, Illinois; Northwestern University, Chicago, Illinois; Boston Medical Center, Gloucester, Massachusetts; University of Massachusetts T.H. Chan School of Medicine, Worcester, Massachusetts; University of Pittsburgh School of Medicine, Pittsburgh, Pennsylvania; Northwestern University, Chicago, Illinois; Northwestern University, Chicago, Illinois; Boston University School of Medicine / Boston Medical Center, Boston, MA

## Abstract

**Background:**

Black/African American and Hispanic/Latino men who have sex with men face a disproportionate burden of new HIV diagnoses. The Keep It Up! (KIU) program, an evidence-based online intervention, is effective in reducing risk behaviors. The KIU 3.0 study, conducted between 2019-2022, compared two delivery strategies: centralized, national direct-to-consumer (DTC) recruitment, and decentralized recruitment via community-based organizations (CBO). Here, we report strategy costs.

**Figure:**
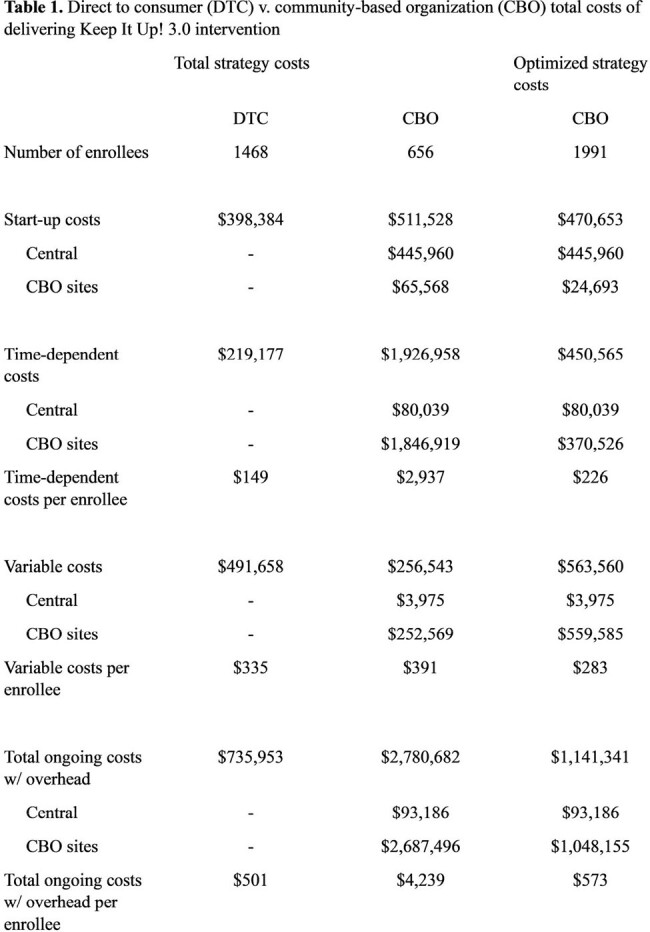

**Methods:**

We conducted interviews to identify and quantify start-up (e.g. equipment, training), time-dependent (e.g. advertisements, payroll), and variable (e.g. incentives, medical materials) costs for both delivery models. We report costs in 2021 USD. We analyzed cost variation among CBOs and present a hypothetical scenario where all CBOs match the efficiency of the two with the lowest cost per participant.

**Figure:**
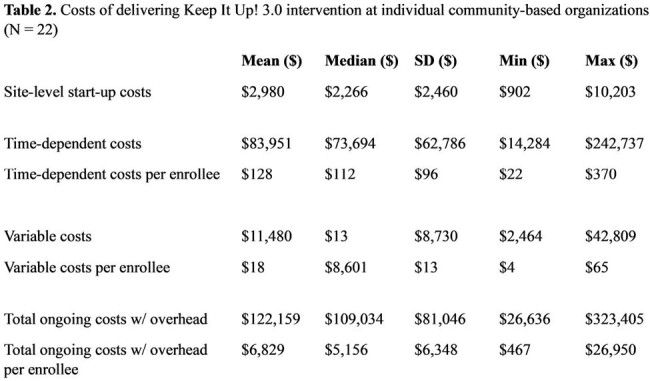

**Results:**

The DTC arm enrolled 1,468 participants. Start-up costs totaled $398,384 of which $376,393 was content design and development. Ongoing costs per participant totaled $501 (66.8% variable, 29.8% time-dependent, and 3.4% overhead, Table 1). The CBO arm enrolled 656 participants across 22 sites. Start-up costs totaled $511,528 of which $401,141 was content design and development. Ongoing costs per participant totaled $4,239 (9.2% variable, 69.3% time-dependent, and 21.4% overhead, Table 1). Site-specific costs ranged from $467 - $26,950 per participant with no correlation to the number of participants (Table 2, Figure 1, Spearman’s Rho = 0.007). In a hypothetical optimized CBO scenario, we estimated that 1,991 participants would enroll, start-up costs would be $470,653 and ongoing costs per participant would be $573, demonstrating that CBO delivery has the potential for similar costs to DTC delivery.

**Figure:**
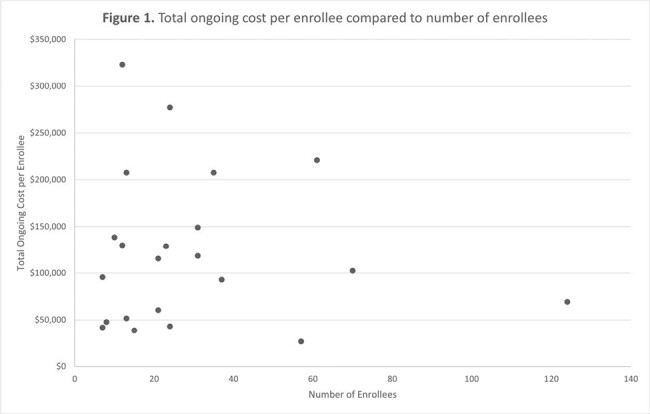

**Conclusion:**

DTC implementation demonstrated lower costs per participant than CBO distribution. CBO costs were highly variable with no correlation to enrollment. It is important to note the challenges related to the COVID-19 pandemic (clinic closures and restrictions, material shortages, and price increases) when contextualizing these costing outcomes. Efficient implementation models are crucial for scaling evidence-based interventions.

**Disclosures:**

**Brian Mustanski, PhD**, Hologic: Grant/Research Support

